# Efficiency of High-Flow Nasal Cannula on Pulmonary Rehabilitation in COPD Patients: A Meta-Analysis

**DOI:** 10.1155/2020/7097243

**Published:** 2020-10-02

**Authors:** Cuiping Fu, Xin Liu, Qingqing Zhu, Xu Wu, Shengyu Hao, Liang Xie, Shanqun Li

**Affiliations:** ^1^Department of Respiratory Medicine, The First Hospital Affiliated to Soochow University, Soochow 215006, China; ^2^Department of Respiratory Medicine, Fujian Province Geriatric Hospital, Fuzhou 350000, China; ^3^Department of Respiratory Medicine, Zhongshan Hospital, Fudan University, Shanghai 200032, China

## Abstract

**Introduction:**

The clinical benefit of high-flow nasal cannula (HFNC) on factors related to pulmonary rehabilitation in chronic obstructive pulmonary disease (COPD) patients remains unclear. This meta-analysis aimed at synthesizing the available evidence on the efficacy of HFNC on exercise capacity, lung function, and other factors related to pulmonary rehabilitation in COPD patients.

**Methods:**

Electronic databases (MEDLINE, Embase, Cochrane Central Register of Controlled Trials, Web of Science) were searched for randomized trials comparing with conventional oxygen therapy (COT) or noninvasive ventilation (NIV). Primary outcomes were respiratory rate, FEV1, tidal volume, oxygen partial pressure, total score of St. George's respiratory questionnaire, 6-minute walk test, and exercise endurance time.

**Results:**

Ten trials met the criteria for inclusion. Combined data from six studies showed that HFNC showed a lower respiratory rate in COPD patients [mean difference -1.27 (95% CI: -1.65–(-0.89)]. Combined data from three studies showed a lower forced expiratory volume in one second (FEV1) in the group of HFNC. No difference in tidal volume was showed between the HFNC and control groups in COPD patients. No significant oxygen improvement between the HFNC groups and control groups. The total score of St. George's respiratory questionnaire was improved by the subgroup analysis of HFNC versus COT but no NIV. Two multicenter RCTs showed the six-minute walk test, and statistical results showed that the length of the six-minute walk capacity was increased after usage of HFNC compared to the control group [mean difference -8.65 (95% CI: -9.12–(-8.19)]. No increase of exercise capacity after usage of HFNC (mean difference -12.65).

**Conclusion:**

In the first meta-analysis of the area, the current evidence did not show so much positive effect on tidal volume or oxygen improvement in COPD patients. Length of the six-minute walk capacity was increased after using HFNC, while other pulmonary rehabilitation parameters, namely, the score of St. George's respiratory questionnaire and exercise capacity show no increase in the group of HFNC. The variance in the quality of the evidence included in this meta-analysis highlights the need for this evidence to be followed up with further high-quality and more randomized trials.

## 1. Introduction

Chronic obstructive pulmonary disease (COPD) is a worldwide cause of mortality with a growing burden [1, 2]. The Global Burden of Disease Study 2015 estimated the global prevalence of COPD at about 174 million cases [3]. In 2010, Adeloye et al. estimated a global prevalence of 384 million cases on the basis of the spirometric criteria in several epidemiological cohorts [4]. It was reported that the overall risk of developing COPD by the age of 80 years has been calculated to be 28%, according to the population-based health administrative data [5]. This disease progressively leads to chronic respiratory insufficiency, which can lead to hypoxia and hypercapnia [6], each of which is associated with poor outcomes [7, 8]. Pulmonary rehabilitation, defined by the American Thoracic Society/European Respiratory Society (ATS/ERS) as a comprehensive intervention based on a thorough patient assessment followed by patient tailored therapies that include, but are not limited to, exercise training, education, and behaviour change, designed by improve physical and psychological condition of people with chronic respiratory disease [9], is a cornerstone in the nonpharmacological management of COPD. Pulmonary rehabilitation has well-established benefits in improving exercise capacity, health-related quality life, and psychological well-being in chronic lung conditions in COPD patients.

Long-term oxygen therapy (LTOT), noninvasive ventilation (NIV), and High-flow nasal cannula (HFNC) oxygen therapy were presented as new protocols of pulmonary rehabilitation of COPD, which may counteract the negative consequences and target modifiable risk factors of COPD patients for hospital readmission. Unfortunately, there are important drawbacks associated with the use of NIV, including interface discomfort, excessive high air pressure, sleep disturbance, and intolerability due to patient-ventilator asynchrony, each of which can lead to poor compliance or treatment failure [10–12]. Therefore, an alternative strategy, HFNC, was warranted.

HFNC oxygen therapy is currently a popular modality of respiratory support in COPD patients. HFNC delivers warmed and humidified oxygen (usually with a blended mix with air) at a higher flow than the patient's inspiratory flow, typically 1-2 L/kg/min [13]. HFNC oxygen therapy is carried out using an air/oxygen blender, active humidifier, single heated tube, and nasal cannula. Able to deliver adequately heated and humidified medical gas at flows up to 60 L/min, it is considered to have a number of physiological advantages compared with other standard oxygen therapies, including reduced anatomical dead space, PEEP, constant FIO2, and good humidification compared to LTOT and NIV. Observational studies suggest HFNC may improve the effects on exercise (showed by constant workload exercise testing) compared with oxygen [14]. It could be a potential manner for improving pulmonary rehabilitation of COPD patients. A recent meta-analysis showed that HFNC reduced PaCO2—an acute exacerbation in stable COPD patients and improved quality of life showed by Saint George's Respiratory Questionnaire. Could HFNC also be an effective pulmonary rehabilitation manner for COPD patients?

Therefore, the overall aim of this meta-analysis was to summarize the available evidence assessing the effects of delivering air or oxygen via HFNC compared with LTOT or NIV and to evaluate the effect of HFNC on pulmonary rehabilitation in COPD patients.

## 2. Methods

### 2.1. Participants

Adults with a confirmed diagnosis of COPD (in line with the national or international criteria, e.g., British Thoracic Society, American Thoracic Society/European Respiratory Society, and Global Initiative for Chronic Obstructive Lung Disease).

### 2.2. Intervention

Studies were included if patients were randomized to HFNC treatment.

### 2.3. Comparison

The comparator was any control group or NIV.

### 2.4. Outcomes

Primary outcomes were respiratory rate, FEV1, tidal volume, oxygen partial pressure, St. George's respiratory questionnaire, six-minute walk test, and exercise endurance time.

### 2.5. Study Design

Studies included in this meta-analysis had to have adhered to the following study designs: parallel-group randomized controlled trials (allocation at the individual or cluster level or using the quasi-random method) or prospective cohort trials or crossover randomized controlled trials (data up to the point to crossover only).

### 2.6. Search Strategy

We searched for publications and abstracts on PubMed, the Cochrane database of systematic reviews, web of science, and Embase, using the search terms on Pubmed: (“HFNC” or “high-flow nasal cannula” or “high-flow nasal therapy” or “high-flow nasal oxygen” or “high-flow therapy”) AND (“pulmonary disease, chronic obstructive”[MeSH Terms] OR COPD[Text Word]). We limited the search to English publications; we did not limit the search based on publication type. We searched both for bench studies and adult human studies. We considered only studies defining nasal highflow as a flow rate ≥ 20 L/min. Search results were collated using NoteExpress (China). Duplicate citations were removed prior to independent screening of title and abstracts according to the inclusion criteria by two reviewers and the detailed selection method referred to the published source [15]. Full-text articles were obtained from all studies that were unable to be excluded based on title and abstract, before further independent screening to decide on final eligibility. Discrepancies in study eligibility were resolved through discussion between reviewers.

### 2.7. Study Selection and Data Extraction

Two independent investigators were assigned to extract the data from the eligible studies by screening titles and abstracts and reviewing full texts. Data from the included studies were recorded in a standard form recommended by Cochrane [16]. We contacted the corresponding or first author to request the subgroup data specifically for the crossover study or if there were any missing data. Any disagreement was resolved by mutual consensus in the presence of all investigators.

### 2.8. Quality Assessment

For the assessment of risk of bias in estimating the study outcomes, we used the Cochrane risk of bias tool [16]. Each study was assessed for (1) random sequence generation (selection bias), (2) allocation concealment (selection bias), (3) blinding of participants and personnel (performance bias), (4) blinding of related outcomes assessment (detection bias), (5) incomplete outcome data (attrition bias), (6) selective reporting (reporting bias), and (7) other biases. Two investigators conducted the quality assessment for the study methodology independently and in duplicate. Any divergence was resolved by mutual consensus in the presence of a third investigator.

### 2.9. Statistical Analysis

All analyses were performed using Review Manager Version 5.3 (Cochrane Collaboration, United Kingdom). Summary estimate of continuous data was presented as weighted mean difference with 95% CI. Statistical heterogeneity was determined using the *Q* test at a level of *α* < 0.1, which measures the extent of inconsistency among results of study (with *I*^2^ values of 25%, 50%, and 75% indicates low, moderate, and high heterogeneity, respectively) [15]. The random effect model was adopted to make analysis when the *P* value less than or equal to 0.1. Otherwise, the fixed effect model was applied. The pooled effect was performed by *Z* test. *P* value less than 0.05 was considered statistical significance.

## 3. Results

### 3.1. Selected Studies and Characteristics

**Figure 1:**
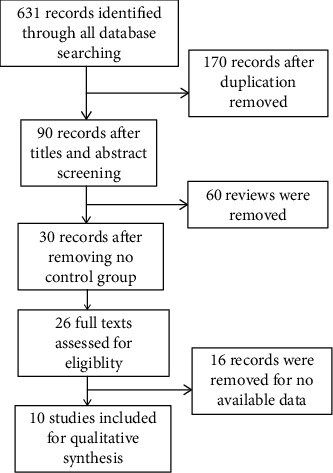
Flow of information through the different phases of the meta-analysis.

Of the 931 citations identified and reviewed, 10 randomized trials of HFNC met the criteria for inclusion (Figure 1). After removing the duplicated papers, we found 867 studies, and 26 trials are relevant with our purpose. Finally, ten RCTs meet our main requirements [17–26]. Sixteen RCTs were excluded because they lacked data on key end points. We have written to the authors to ask the key data but receive no results. All cohort studies were excluded. Primary outcomes were lung function and six-minute walk test. Studies were eligible for inclusion if they involved an RCT using HFNC as a treatment method in COPD patients, provided they reported at least one of the following outcomes: respiratory rate, FEV1, tidal volume, oxygen partial pressure, St. George's respiratory questionnaire, 6-minute walk test, and exercise endurance time.  Table 1 Summarizes the characteristics of each individual study.

Each trial enrolled had clear criteria to decide the inclusion and exclusion of participants. Participants of the two groups were comparable in each trial enrolled. The included trials randomized between 12 and 200 patients were mostly RCTs except two trials [20, 21], and two of them were multicenter RCTs [22, 23]. The COPD types were stable COPD except one trial reported stable and exacerbated COPD patients. The mean ages of the trials showed that the effect of HFNC on elder people was analyzed in this meta-analysis. For the control group, two trials reported noninvasive mechanical ventilation, and the other trials were conventional oxygen therapy (COT) [27]. Among these 10 studies, 6 studies showed results of respiratory rate, 3 studies showed results of FEV1, 4 studies showed results of tidal volume, 6 studies showed results of the oxygen partial pressure, 3 studies showed results of St. George's respiratory questionnaire, 2 studies showed results of 6-minute walk test, and 2 studies showed results of exercise endurance time.

### 3.2. Quality Assessment

**Figure 2:**
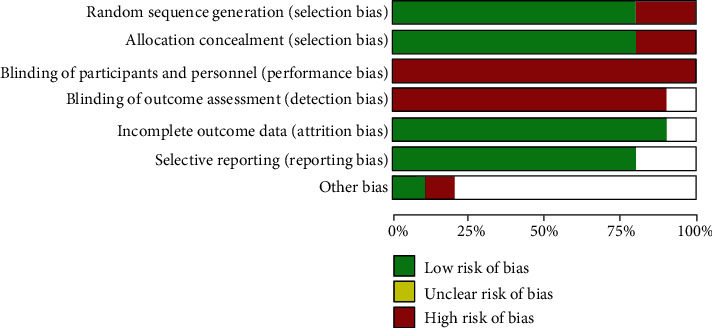
Methodological quality and risk of bias analyzed by the Cochrane Risk of Bias Tool.

For the assessment of methodological quality and risk of bias (RoB) about the included studies, we used the Cochrane Risk of Bias Tool that classifies RoB as “low,” “high,” or “unclear” for seven aspects mentioned in the part of methods. Other bias included detailed determination of COPD and detailed follow-up. Each item scored “low risk” if reported, “high risk” if not reported, or “unclear risk” if no adequate information is available in the article. The ROB of each eligible trial was shown in Figure 2.

### 3.3. Unit of Analysis Issues

HFNC reduces minute volume, lowers respiratory rate, and decreases the work of breathing. The respiratory rate was reduced in HFNC only. The 6MWT distance increased with both devices, although this was not significant for HFNC.

**Figure 3:**
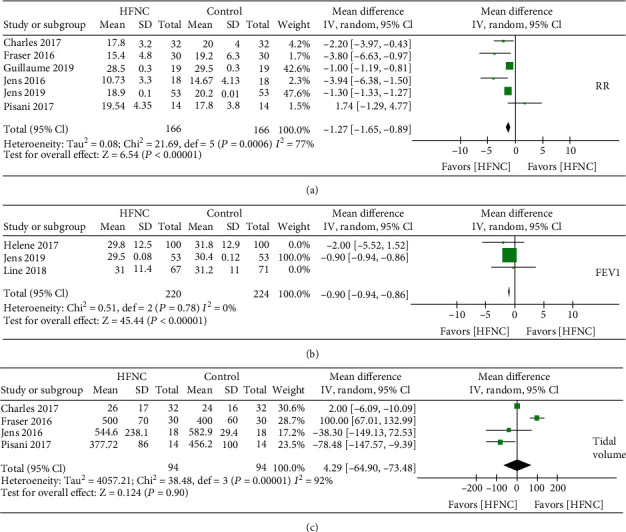
(a) Forest plot of mean difference of the respiratory rate in HFNC arm compared with control arm. (b) Forest plot of mean difference of FEV1 in HFNC arm compared with control arm. (c) Forest plot of mean difference of tidal volume in HFNC arm compared with control arm.

**Figure 4:**
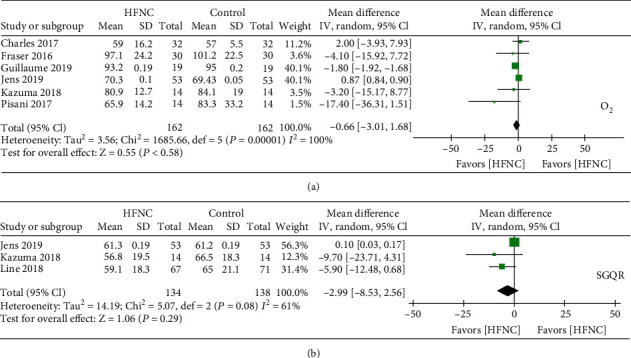
(a) Forest plot of mean difference of the oxygen partial pressure in HFNC arm compared with control arm. (b) Forest plot of mean difference of total score of St. George's respiratory questionnaire in HFNC arm compared with control arm.

**Figure 5:**
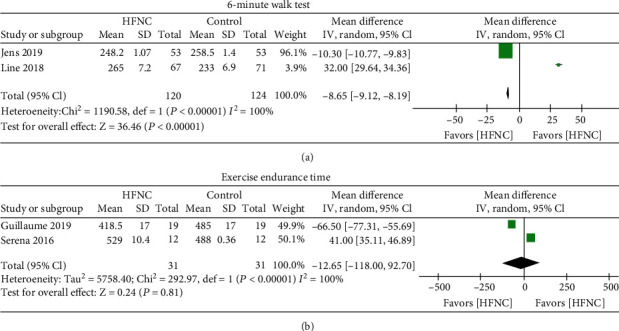
(a) Forest plot of mean difference of the 6-minute walk test in HFNC arm compared with control arm. (b) Forest plot of mean difference of exercise endurance time in HFNC arm compared with control arm.

Six studies provided information on the respiratory rate; the combined data of these studies showed that HFNC showed a lower respiratory rate in COPD patients [mean difference -1.27 (95% CI: -1.65–(-0.89)] (Figure 3(a)), with high heterogeneity in the data (chi^2^ = 21.69, *I*^2^ = 77%, *P* < 0.00001). Several studies reported lung function after treatment of HFNC or normal low flow oxygen. Three studies provided information on FEV1 and tidal volume. The combined data of these studies showed lower FEV1 in the group of HFNC compared to the control (chi^2^ = 0.51, *I*^2^ = 0%, *P* < 0.00001, Figure 3(b)). The combined data of these studies showed no difference in tidal volume between HFNC and control groups in COPD patients (chi^2^ = 38.48, *I*^2^ = 92%, *P* < 0.00001, Figure 3(c)]. The oxygen partial pressure in arterial blood gas analysis was reported in six clinical trials, and combined studies did not show a significant difference in oxygen improvement between HFNC groups and control groups (Figure 4(a)). However, subgroup analysis of HFNC versus COT but not NIV (discarding Jens 2019 and Pisani 2017 in the subgroup analysis) showed an elevation of oxygen pressure in COPD patients. Quality of life showed by St. George's respiratory questionnaire was reported in three trials. However, the combined data did not show HFNC could improve the total score of St. George's respiratory questionnaire (Figure 4(b)). The total score of St. George's respiratory questionnaire was improved in the subgroup analysis of HFNC versus COT but not NIV (discarding Jens 2019 and Pisani 2017 in the subgroup analysis). Data also showed the exercise capacity after HFNC or control treatment. Two multicenter RCTs showed the six-minute walk test, and statistical results showed that the length of the six-minute walk capacity was increased after usage of HFNC compared to the control group [mean difference -8.65 (95% CI: -9.12–(-8.19)] (Figure 5(a)), with high heterogeneity in the data (chi^2^ = 1190.98, *I*^2^ = 100%, *P* < 0.00001). Two RCTs showed the exercise endurance time, and statistical results showed no increase of exercise capacity after usage of HFNC compared to the control group [mean difference -12.65] (Figure 5(b)).

### 3.4. Sensitivity Analysis

The sensitivity analysis of six studies reporting respiratory rates after HFNC showed that discarding the article of the article of Guillaume et al. [19] will decrease the heterogeneity by 9%. The sensitivity analysis of four studies of tidal volume showed that discarding the article of Fraser et al. [18] will decrease the heterogeneity by 28%. The sensitivity analysis of six studies of oxygen partial pressure showed that discarding the article by Guillaume et al. [19] or Jens [20] will decrease the heterogeneity by 83% or 91%, respectively. The sensitivity analysis of three studies of the total score of St. George's respiratory questionnaire showed that the article of Jens 2019 was the main source of total heterogeneity by 61%. Discarding of this article, the results would support that HFNC improve the quality life questionnaire.

## 4. Discussion

There were previous systematic reviews and meta-analyses in COPD-related pulmonary rehabilitation [28], and the most available evidence was about pulmonary exercise programs or muscle exercise. HFNC, as a new therapeutic manner in COPD patients with high efficiency compared to COT and high adherence rate and comfortable experience compared to NIV, emerged as a new method for pulmonary rehabilitation in COPD patients. To our knowledge, this is the first meta-analysis to identify ten completed randomized trials that compared the efficacy of HFNC on COPD patients, especially in the aspect of pulmonary rehabilitation. High-flow therapy accompanied with a higher tidal volume and improved inspiratory flow dynamics improves oxygenation and provides adequate minute ventilation and the lower rate of reintubation or NIV secondary to hypoxia in the HFNC group [29, 30]. Most primary outcome was negative in this meta-analysis. We pointed out that HFNC was not significant in improving FEV1, tidal volume, PO2, SGQR, and exercise endurance time in COPD patients.

FEV1 was showed lightly decreased in the group of HFNC, while only three RCTs reported this data. Considering no significant difference in tidal volume after usage of HFNC, HFNC could not improve the lung function compared to COT or NIV. HFNC could improve the oxygen partial pressure compared to COT but not NIV. Other meta-analyses like Bonnevie et al. [31] comparing HFNC and low-flow oxygen therapy demonstrated that HFNC reduced PaCO2, an acute exacerbation and improved quality of life in stable COPD patients, and also suggested that HFNC did not improve SpO2 both at short- and long-term. The latest meta-analysis by Tristan et al. [28] supported the use of NHFC to treat acute respiratory failure in COPD patients, but not significantly improved exercise capacity, hospitalization rate, or mortality. However, our meta-analysis did not demonstrate a significant increased exercise capacity with HFNC.

The ability of HFNC to reduce the respiratory rate was consistent with a reduction in the work of breathing. The mechanism is most likely the reduced anatomical dead space assisted by the positive expiratory pressure effect of HFNC, which allows for improved ventilation and perfusion matching [32]. Additionally, matching the inspiratory flow demands with HFNC overcomes nasopharyngeal inspiratory resistance.

The six-minute walk test was reported with a longer time in the group of HFNC, which indicated an improved exercise capacity of HFNC. The ix-minute walk test is a parameter of evaluating exercise capacity. The lung function test was reported to evaluate the improvement of pulmonary rehabilitation. It was reported that six weeks of treatment with HFNC therapy improved health-related quality of life and reduced hypercapnia in patients with stable COPD [23]. However, this meta-analysis included only two trials about exercise endurance time with high heterogeneity. Only one study mentioned minute ventilation [17], and no difference was showed between HFNC and the control group. This meta-analysis could not provide enough evidence that HFNC improve exercise capacity in COPD patients.

Although the randomized and controlled study method was used in eight trials, other potential confounding factors and bias could not be avoided as a double-blind investigation was not conducted in most of the included trials. Level of quality in the present meta-analysis ranged from moderate to high. Limitations include lack of sufficient data to explore relevant subgroup effects, inclusion of some cohort and crossover trials, and outcome data affected by inconsistence and imprecision for most outcomes. Besides, there was high clinical heterogeneity in terms of some primary outcome.

Our findings have general applicability to HFNC referred to pulmonary rehabilitation in COPD patients. As none of the included trials stratified randomization by COPD severity, it is unclear whether our findings are equally applicable to all stages of COPD severity or exacerbation status. Further research is required to ascertain the effects of HFNC on lung function and exercise capacity. Parallel RCTs are needed to confirm the present results and provide more data on patient-centered outcomes such as quality of life, exacerbation, and hospitalization.

## Figures and Tables

**Table 1:** Detailed characteristic of the ten enrolled studies.

Author (year)	Population (H, C)	Age	Country	Study design	NCT	COPD type	Control	Length of therapy	Main outcomes^a^
Atwood (2017) [17]	32 (16, 16)	69 (9)	United States	S, RCT	NCT00990210	Stable	LTOT	20 min	(1), (3), (4)
Fraser et al. (2016) [18]	30 (15, 15)	NR	Australia	RCT crossover	ACTRN12613000028707.	Stable	LTOT	20 min therapy-20 min washout -20	(1), (3), (4)
Guillaume et al. (2019)[19]	19 (19, 19)	62.1 (9.1)	French	S, RCT, crossover	NCT03058081	Stable	Room air or oxygen	3 days including washout	(1), (4), (7)
Helene et al. (2017) [20]	100 (50, 50)	67.2 (8.5)	Germany	Prospective, cohort.	NCT01686893 & NCT01693146	Stable	COT	60 min COT-washout-60 min HFNC	(2)
Jens et al. (2016) [21]	67 (48, 19)	60 (11.4)	Germany	Prospective, cohort.	NCT02504814	Stable	Mechanical treatment	NR	(1), (3)
Jens et al. (2019)[22]	94 (50, 44)	65.3 (9.3)	Germany	M, RCT, crossover	NCT02007772	Stable	NIV	12 weeks including washout	(1), (2), (4), (5), (6)
Kazuma et al. (2018)[23]	32 (16, 16)	73.8 (6.9)	United states	M,RCT, crossover	NCT02545855	Stable hypercapnic	LTOT	12 weeks including washout	(4), (5)
Line et al. (2018)[24]	200 (100, 100)	71 (8.2)	Europe	RCT, crossover	NCT02731872	Stable and acute exacerbation	LTOT	12 months	(4), (5), (6)
Pisani et al. (2017)[25]	14 (14, 14)	NR	Italy	Random, crossover	NCT02363920	Stable	NIV	150 min	(1), (3), (4)
Serena et al. (2016)[26]	12 (12, 12)	70 (8)	Italy	S,RCT, crossover	NR	Stable severe	Air condition	Three days including washout	(7)

^a^Outcome measures include (1) respiratory rate, (2) FEV1, (3) tidal volume, (4) the oxygen partial pressure, (5) total score of St. George's respiratory questionnaire, (6) 6-minute walk test, (7) and exercise endurance time. NIV: noninvasive ventilation; H: HFNC; C:control. LTOT: long-term oxygen therapy; NCT: National Clinical Trial; S: single center; M: multicenter; RC: randomized controlled trial; NR: not reported; COT: conventional oxygen therapy.

## Data Availability

Data listed in this manuscript could be available as request.

## Supplementary Materials

Supplementary Materials Table S1. The search strategy of database.
