# Supplementary material for: Efficiency of High-Flow Nasal Cannula on Pulmonary Rehabilitation in COPD Patients: A Meta-Analysis
Source: Biomed Res Int. 2020 Oct 2;2020:7097243. doi: 10.1155/2020/7097243 (PMC7556103; doi:10.1155/2020/7097243)
Supplement: Supplementary Materials — Table S1. The search strategy of database. [file 7097243.f1.docx]

Table S1. The search strategy of database.

| Data base | Search strategy | Number of articles (Results) |
| --- | --- | --- |
| Pubmed | (“HFNC” or “high flow nasal cannula” or “high flow nasal therapy” or “high flow nasal oxygen” or “high flow”) AND ("pulmonary disease, chronic obstructive"[MeSH Terms] OR COPD[Text Word]) | 178 |
| Web of science | TS=((HFNC OR "high flow nasal cannula" OR "high flow nasal therapy" or "high flow nasal oxygen" or "high flow") AND ("pulmonary disease chronic obstructive" OR COPD)) | 106 |
| Embase | ('HFNC' OR 'high flow nasal cannula'/exp/mj OR ' high flow nasal oxygen'/exp/mj) AND [1990-2020]/py combined with ('pulmonary disease chronic obstructive'/exp/mj OR ' COPD' /exp/mj) AND [1990-2020]/py search in Embase and medline database | 347 |
